# Sex Differences of Periaqueductal Grey Matter Functional Connectivity in Migraine

**DOI:** 10.3389/fpain.2021.767162

**Published:** 2021-11-30

**Authors:** Kinga Gecse, Daniel Baksa, Dóra Dobos, Csaba Sandor Aranyi, Attila Galambos, Natália Kocsel, Edina Szabó, Gyöngyi Kökönyei, Miklós Emri, Gyorgy Bagdy, Gabriella Juhasz

**Affiliations:** ^1^Department of Pharmacodynamics, Faculty of Pharmacy, Semmelweis University, Budapest, Hungary; ^2^SE-NAP2 Genetic Brain Imaging Migraine Research Group, Hungarian Brain Research Program, Semmelweis University, Budapest, Hungary; ^3^Division of Nuclear Medicine and Translational Imaging, Department of Medical Imaging, Faculty of Medicine, University of Debrecen, Debrecen, Hungary; ^4^Institute of Psychology, ELTE Eötvös Loránd University, Budapest, Hungary; ^5^Center for Pain and the Brain (PAIN Research Group), Department of Anesthesiology, Critical Care and Pain Medicine, Boston Children's Hospital, Harvard Medical School, Boston, MA, United States; ^6^NAP-2-SE New Antidepressant Target Research Group, Hungarian Brain Research Program, Semmelweis University, Budapest, Hungary; ^7^MTA-SE Neuropsychopharmacology and Neurochemistry Research Group, Hungarian Brain Research Program, Semmelweis University, Budapest, Hungary

**Keywords:** headache, periaqueductal grey (PAG), male, female, pain intensity, disability

## Abstract

The existence of “sex phenotype” in migraine is a long-standing scientific question. Fluctuations of female sex hormones contribute to migraine attacks, and women also have enhanced brain activity during emotional processing and their functional brain networks seem to be more vulnerable to migraine-induced disruption compared to men. Periaqueductal grey matter (PAG) is a core region of pain processing and modulation networks with possible sex-related implications in migraine. In our study, sex differences of PAG functional resting-state connectivity were investigated in the interictal state in 32 episodic migraines without aura patients (16 women and 16 men). A significant main effect of sex was detected in PAG connectivity with postcentral, precentral, and inferior parietal gyri, and further differences were found between right PAG and visual areas (superior occipital gyrus, calcarine, and cuneus), supplementary motor area, and mid-cingulum connectivity. In all cases, PAG functional connectivity was stronger in female migraineurs compared to males. However, higher average pain intensity of migraine attacks correlated with stronger connectivity of PAG and middle temporal, superior occipital, and parietal gyri in male migraineurs compared to females. Migraine-related disability is also associated with PAG connectivity but without sex differences. Our results indicate that sex differences in PAG connectivity with brain regions involved in sensory and emotional aspects of pain might contribute to the “sex-phenotype” in migraine. The stronger functional connectivity between PAG and pain processing areas may be a sign of increased excitability of pain pathways even in resting-state in females compared to male migraineurs, which could contribute to female vulnerability for migraine. However, pain intensity experienced by male migraineurs correlated with increased connectivity between PAG and regions involved in the subjective experience of pain and pain-related unpleasantness. The demonstrated sex differences of PAG functional connectivity may support the notion that the female and male brain is differently affected by migraine.

## Introduction

Despite the extensive research in recent decades and successful drug developments, migraine remains one of the major causes of disability worldwide and the first cause among young women (1). Migraine affects women three times more than men suggesting that sex differences, among other factors, take part in migraine attack generation (2). Pain perception and pain-related unpleasantness alter during the menstrual cycle (3), and hormonal fluctuations make women more sensitive to migraine triggers (4, 5). Furthermore, perimenopausal women with migraine have a higher allostatic load than non-migraineurs (6) that leads to maladaptive stress responses and reinforces the generation of migraine attacks (7).

In addition to hormonal factors, recent structural and functional imaging studies revealed that the brains of women and men are differentially affected by migraine (8). A meta-analysis reported a decreased grey matter volume in the right dorsolateral prefrontal cortex among female migraineurs (9). Moreover, increased cortical thickness in insula and precuneus was found in female migraineurs compared to males and healthy controls from both sexes (10). In addition, stronger functional connectivity of these two regions with other emotional processing areas (e.g., primary somatosensory cortex) was observed in female migraineurs suggesting that females seem to be more vulnerable to migraine-induced disruption in emotional circuitry (10). Migraine patients showed enhanced somatosensory response to emotional stimuli in a face emotional task indicating an increased sensitivity to psychosocial stressors (11). Disrupted functional connectivity in brain regions involved in sensory processing and pain modulation has been also demonstrated among female migraineurs compared to healthy women (12), but there was no comparison with males. Migraine is considered a typical disease in women and they are overrepresented in migraine research. However, recent findings pointed out the importance of involving men in migraine studies to distinguish male- and female-specific neuropathology (8, 13).

A possible candidate region for detecting further cerebral differences between female and male migraineurs is the periaqueductal grey matter (PAG), a core region of a pain processing and modulating network. First, PAG dysfunction contributes to migraine pathogenesis (14), wherein the increased activity of PAG was detected during migraine attacks (15) and altered interictal PAG functional connectivity (PAG-FC), with nociceptive and somatosensory processing pathways, was also found (16). Second, sex differences were revealed in pain-induced PAG connectivity in non-migraineurs (13), specifically, men who had increased PAG-FC with the amygdala, putamen, and caudate compared to women. This result obtained from a pain task may suggest that PAG connectivity may also differ between sexes among migraine patients. Finally, female hormones influence prostaglandin E2 (PGE_2_) and calcitonin gene-related peptide (CGRP) levels in PAG (17), representing a mechanism that could contribute to migraine attack development in women.

Based on the above findings, we hypothesised that PAG functional connectivity may show differences between female and male migraineurs. In addition, we assumed that sex might have an impact on the relationship between PAG connectivity and migraine attack frequency and migraine-related disability. To test this hypothesis, we conducted a resting-state fMRI study with migraine patients.

## Materials and Methods

### Participants

Thirty-two age-matched episodic migraine patients without aura were included in the study, 16 men (mean age = 29.73 ± 6.12 years) and 16 women (mean age = 29.36 ± 6.20 years). To achieve an adequate sample size, data from two studies were pooled together. Participants were pain- and medication-free 48 h before the examination and did not use any daily medication, except contraceptives. Before entering the study, the mental health of participants was checked by senior researchers using the Mini-International Neuropsychiatric Interview (18) and episodic migraine without aura was diagnosed by expert neurologists using International Classification of Headache Disorders-III criteria (19). Participants did not have any past or current serious medical, neurologic, or psychiatric disorders. They were all right-handed in accordance with the Edinburgh Handedness Inventory (20). On the fMRI examination day, participants completed the Migraine Disability Assessment (MIDAS) questionnaire (21) and were asked about the frequency of migraine attacks per month.

The study protocol was approved by the Scientific and Research Ethics Committee of the Medical Research Council (Hungary). The entire study was conducted according to the Declaration of Helsinki and with the written informed consent of each participant.

### Imaging Data Acquisition

Resting-state fMRI was obtained using two different types of 3 Tesla MRI scanners: half of the participants (eight women and eight men) was scanned with an Achieva 3 T Philips Medical System (Philips Healthcare, Amsterdam, Netherlands), while the other half of our sample with a 3 T MAGNETOM Prisma Siemens Syngo (Siemens Healthineers, Erlangen, Germany). The study protocol and the imaging data preprocessing steps were the same in both cases. Participants were required to close their eyes, but remain awake. The imaging dataset acquisition parameters of Philips Achieva T2^*^-weighted echo-planar (EPI) pulse-sequence were as follows: repetition time (TR) = 2,500 ms, echo time (TE) = 30 ms, field of view (FOV) = 240 mm^2^; with a 3 × 3 × 3 mm resolution. The imaging dataset acquisition parameters of MAGNETOM Prisma were as follows: TR = 2,220 ms, TE = 30 ms, FOV = 222 mm^2^, with a 3 × 3 × 3 mm resolution. High-resolution (1 × 1 × 1 mm) structural data were acquired before the resting-state session using a T1-weighted 3D turbo field echo (TFE) sequence with Philips scanner and 3D MPRAGE sequence with Siemens scanner. The possible effect of the scanners was taken into account during the statistical analysis.

### Self-Reported Measures

The first five items of the MIDAS questionnaire (21) were summed and used as a measure of migraine-related disability in the last 3 months before the experiment. The MIDAS-B (“*On a scale of 0–10, on average how painful were these headaches?”*) was used to measure the average pain intensity of migraine attacks. Two participants (one female and one male) did not complete the MIDAS questionnaire.

### Data Analysis

The characteristics and sex differences of participants in self-reported data were analysed using SPSS (IBM Corp., Armonk, NY, USA, SPSS Statistics for Windows, Version 27) with a significance threshold set at *p* < 0.05.

### Image Preprocessing

Freesurfer version 5.3 (https://surfer.nmr.mgh.harvard.edu/) (22) was used to perform non-uniformity correction, intensity normalisation, and segmentation of the structural images to extract the brain volume and to separate white matter and grey matter tissues from cerebrospinal fluid (CSF). Spatial normalisation to a 2 mm isovoxel brain template in MNI152 space (Montreal Neurological Institute, McGill University, Montreal, Canada) (23) was performed with the advanced normalisation tools (ANTs) registration tool (24). Motion correction FMRIB's linear image registration tool (MCFLIRT) (25) utility of the FMRIB's Software Library (FSL version 6 created by the Analysis Group, FMRIB, Oxford, UK) was used for primary motion correction. In the spatial standardisation step, the FMRIB's Software Library (FSL) linear registration tool coregistered the functional images with the structural image of the corresponding subject, then transformed them into MNI152 space. CompCor technique (26) was used to compute the first five principal components of the fMRI time-series within the white matter and CSF. Spatial filtering was performed with the FSL application SUSAN (27) using a 6 mm Gaussian kernel. Independent component analysis based on automatic removal of motion artefacts (ICA-AROMA) (28) was used to remove signal components classified as related to motion. For further signal correction, we regressed nuisance variables of 24 movement-related effects computed from rigid body transformation parameters (29), and the five principal components amount to the most variation measured in selected noise regions (white matter and cerebrospinal fluid) (26), which were segmented by Freesurfer. Accounting for white matter and CSF signals is especially important in the investigation of PAG, which is highly influenced by the nearby sources of noise. Finally, temporal band-pass filtering in the range of 0.009 and 0.08 Hz was applied. The schematic picture of preprocessing steps is provided in the Supplementary Figure 1.

### Resting-State fMRI Analysis

Seed-to-voxel analysis was conducted with left and right PAG as seed regions defined after Mainero et al. (16) (Left PAG = −2; −28; −6; Right PAG = 4; −28; −6; coordinates in MNI space with a 3 mm radius; see Supplementary Figure 2). Seed-based connectivity maps were generated by voxel-wise Pearson correlation with averaged seed region data of each subject and converted into *Z*-scores using Fisher transformation.

These individual connectivity matrices were entered to a second-level full factorial ANOVA using Statistical Parametric Mapping (SPM12) software package (Wellcome Department of Imaging Neuroscience, Institute of Neurology, London, UK; http://www.fil.ion.ucl.ac.uk/spm12/) implemented in Matlab 2016a (Math Works, Natick, MA, USA). An explicit grey matter mask was applied in every analysis for noise-reduction, the template was provided by the Brain Imaging Centre (Montreal Neurological Institute, McGill University: https://digital.lib.washington.edu/researchworks/handle/1773/33312). To be able to evaluate the scanner effect the first factor in the full factorial ANOVA was scanner type. The second factor was sex and we investigated the main effect of the scanner, the main effect of sex, and their potential interaction. To follow up the ANOVA results, *post-hoc t*-tests were performed.

The main effect of migraine disability, average pain intensity, and migraine frequency, and their interaction effect with sex on PAG intrinsic functional connectivity (PAG-FC) were investigated with two-sample *t*-tests.

To investigate the menstrual cycle differences on PAG-FC in female participants, the PAG-FC of females in a luteal phase were compared to PAG-FC of females in the follicular phase using two-sample *t*-tests. The effect of oral contraceptives on PAG-FC was also investigated in a two-sample *t*-test comparing the group of females using birth control pills to those who are not using them.

All models were corrected for motion parameters and age. We report peak *T*-values for our analysis in clusters where family-wise error corrected *p*_FWE_ < 0.05 significance threshold were reached in at least 20 contiguous voxels (30).

## Results

### Participant Characteristics

The men and women migraine group did not differ in age, migraine frequency, average pain intensity, or MIDAS total score (refer to Table 1), which means that their migraine severity was similar. The number of participants was equally distributed between the two types of MR scanners, 16 participants (eight women) were scanned in each. Among female participants, six used oral contraceptives. Among those who were not on contraceptives, six were in the luteal phase and four were in the follicular phase. There was no difference in the characteristics of the participants according to the scanner site (refer to Supplementary Table 1).

**Table 1:** Characteristics of the participants.

	**Male**	**Female**	**Test statistics (*U*)**	***p***
Number of participants	16	16		
Age (years)	29.73 ± 6.12	29.36 ± 6.20	119.5	0.747
Migraine frequency / month	3.34 ± 2.23	2.80 ± 3.17	92.5	0.176
MIDAS total score	7.93 ± 7.42	11.6 ± 12.32	99.0	0.574
Average pain intensity (MIDAS-B)	5.20 ± 1.96	5.73 ± 1.44	99.5	0.578

*Data are expressed as mean value ± SD. The p-values are based on Mann-Whitney U-tests. MIDAS: Migraine Disability Assessment questionnaire, MIDAS total score was calculated as a measure of migraine-related disability, MIDAS-B refers to the average pain intensity of migraine attacks*.

### Main Effects of Sex on PAG-FC

**Figure 1:**
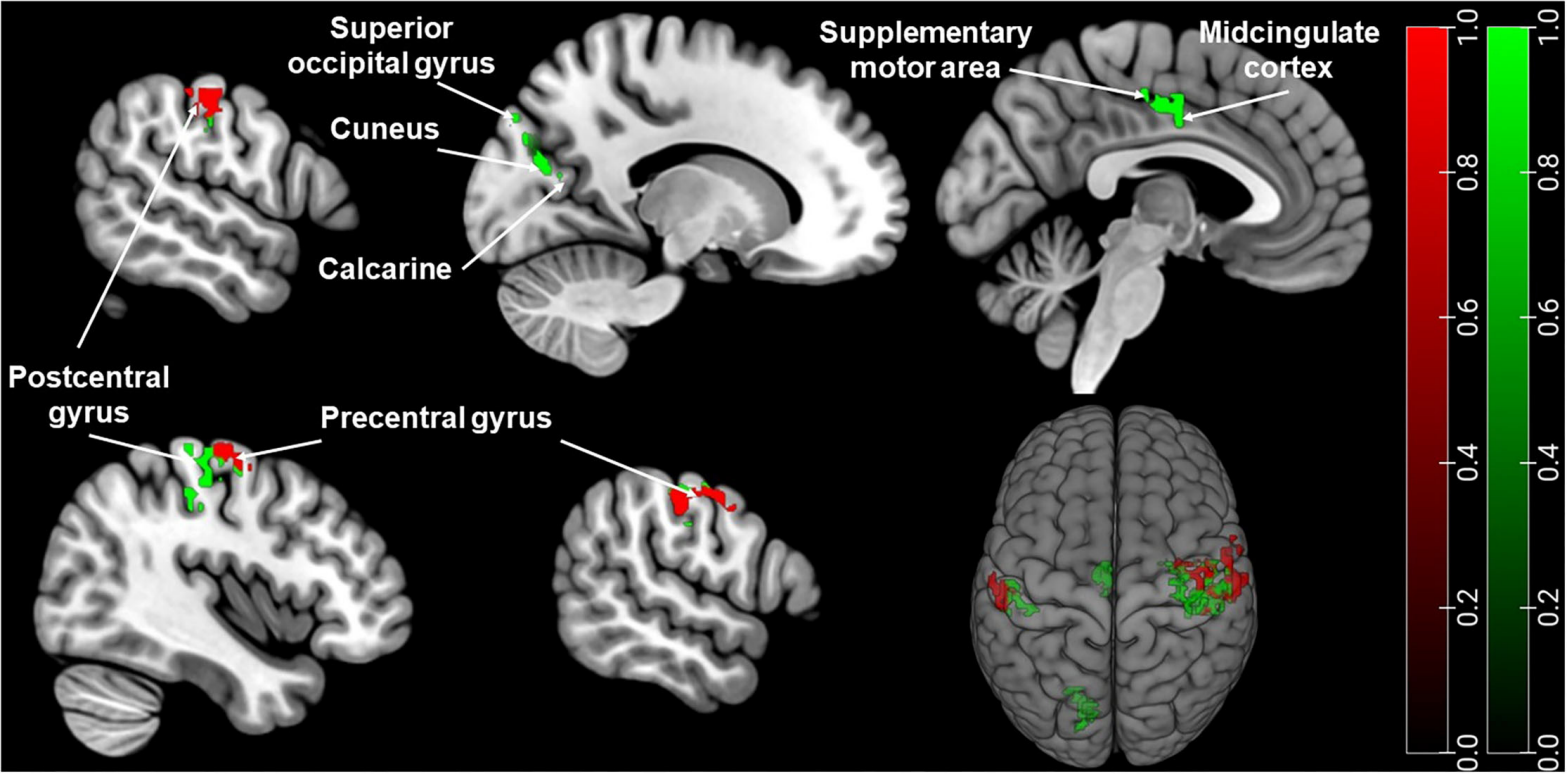
Brain regions with significantly different PAG-FC between men and women. Green: right PAG connectivity, Red: left PAG connectivity. The significance threshold was set at cluster-level *p*_FWE_ < 0.05. Coordinates are in Montreal Neurological Institute (MNI) space. For visualisation, the thresholded statistical map generated by SPM was added as an overlay image on the MNI152 brain template image in MRIcroGL (http://www.mccauslandcenter.sc.edu/mricrogl/) (31).

Sex showed a significant main effect in ANOVA analysis on PAG-FC with postcentral gyrus, precentral gyrus. Sex also had a significant effect on the left PAG-FC with the inferior parietal lobe. In the case of right PAG, sex had an additional significant effect on PAG-FC with calcarine, cuneus, superior occipital gyrus, supplementary motor area (SMA), and midcingulate cortex (MCC) (refer to Figure 1 and Supplementary Table 2).

A *post-hoc t*-test revealed a significantly stronger PAG-FC in female migraineurs compared to males with the above-mentioned regions. There was no stronger PAG-FC in males compared to females (refer to Table 2).

**Table 2:** Brain regions showing a significantly stronger PAG–FC in female migraineurs compared to males.

**Cluster size** **(voxel)**	**Region**	**Peak-coordinates**			**Peak *T*-value**
		**x**	**y**	**z**	
**Left PAG**					
217	R Midcingulate cortex	4	−12	46	6.41
	R Supplementary motor area	6	6	54	4.85
357	R Postcentral gyrus	60	−14	42	5.71
	R Precentral gyrus	64	8	28	4.96
204	L Inferior parietal gyrus	−56	−20	50	5.49
	L Postcentral gyrus	−54	−28	54	4.68
220	R Precentral gyrus	40	−10	62	5.44
	R Postcentral gyrus	48	−20	60	4.53
**Right PAG**					
859	R Postcentral gyrus	60	−14	42	6.29
	R Precentral gyrus	40	−12	64	5.97
160	L Calcarine	−20	−70	14	5.94
	L Cuneus	−14	−76	24	5.74
	L Superior occipital gyrus	−14	−86	40	4.69
112	R Calcarine	22	−66	4	5.62
	R Cuneus	16	−76	24	4.43
202	L Postcentral gyrus	−54	−20	52	5.28
158	L Paracentral_Lobule	−10	−24	52	5.04
	L Supplementary motor area	−8	−12	48	4.78
	L Midcingulate cortex	−4	−6	38	4.50
111	R Supplementary motor area	8	−14	56	4.79
	R Midcingulate cortex	12	−20	44	4.41

*Reported results are significant at cluster-level p_FWE_ < 0.05. Coordinates are in Montreal Neurological Institute (MNI) space. R, right hemisphere; L, left hemisphere*.

The type of the MR scanner had no main effect on PAG-FC and no interaction was revealed between sex and MR scanner.

### Migraine Characteristics and PAG-FC

Higher migraine disability showed a significant correlation with lower PAG and superior medial part of frontal gyrus connectivity. However, there were no differences between male and female migraineurs in the effect of migraine disability on PAG-FC (see Table 3).

**Table 3:** Associations of migraine frequency, migraine disability, and average pain intensity with PAG-FC.

**Cluster size** **(voxel)**	**Region**	**Peak-coordinates**			**Peak *T*-value**
		**x**	**y**	**z**	
**Migraine disability**					
**Negative correlation**					
**Left PAG**					
91	R Superior medial part of frontal gyrus	4	54	26	5.48
**Right PAG**					
109	R Superior medial part of frontal gyrus	4	54	26	5.06
**Average pain intensity**					
**Male>** **Female**					
**Left PAG**					
200	R Superior occipital gyrus	34	−78	46	5.05
	R Superior parietal gyrus	26	−60	50	4.82
	R Angular gyrus	36	−66	40	4.72
**Right PAG**					
138	R Middle occipital gyrus	48	−76	30	4.06
	R Middle temporal gyrus	54	−72	18	4.03
	R Angular gyrus	42	−56	24	3.72

*Reported results are significant at cluster-level p_FWE_ < 0.05. Coordinates are in Montreal Neurological Institute (MNI) space. Migraine disability was assessed with the total score of MIDAS (Migraine Disability Assessment questionnaire). The average pain intensity of migraine attacks was measured by MIDAS-B. R, right hemisphere; L, left hemisphere*.

Average pain intensity showed no main effect, but a significant interaction with sex on PAG-FC. In male migraineurs, a significant positive correlation was found between pain intensity and PAG functional connectivity with angular gyrus, right superior parietal and occipital gyri in case of left PAG, and with angular, middle occipital, and temporal gyri in case of right PAG (refer to Table 3).

Migraine frequency did not show a significant association with PAG connectivity and there was no difference between male and female groups.

### Effect of Female Hormones on PAG-FC

There were no significant differences in PAG-FC between female migraineurs using oral contraceptives compared to those who were not using. There was no significant difference in PAG-FC between women in the luteal phase compared to females in the follicular phase.

## Discussion

In this study, resting-state PAG functional connectivity (PAG-FC) was compared between female and male migraine patients to gain new insights into sex-related differences of the “migraine brain.” Some important migraine-related characteristics (attack frequency, migraine-related disability, and average pain intensity) were also taken into account to achieve a more specific exploration of the mechanisms involved in altered PAG-FC in migraine in general, and specifically, in association with sex.

For the first time, we were able to identify sex-related differences in PAG-FC among migraineurs in the interictal state: female migraineurs compared to males showed a stronger resting-state PAG-FC with somatosensory and motor cortex, visual areas network (superior occipital gyrus, calcarine, cuneus), SMA and MCC. Among the measured migraine characteristics, only the average pain intensity interacted with sex on PAG-FC: higher pain intensity correlated with stronger PAG-FC with middle temporal gyrus, superior occipital and parietal gyrus in male migraine patients compared to females. Migraine-related disability is also associated with PAG-FC but without sex differences.

### Sex-Related Differences in PAG-FC

Sex showed a main effect on PAG –FC with many regions of bottom-up pain processing (32). The ascending pain pathways including somatosensory and motor cortices are involved in pain perception (33), thus the increased PAG-FC with these regions may contribute to pain facilitation and central sensitisation in female migraineurs.

Further analyses revealed that PAG-FC with somatosensory and motor cortices were significantly stronger among female migraineurs compared to males. Our results are in concordance with the Mainero et al. study, where they also detected increased connectivity between PAG and somatosensory and motor cortex in migraine patients who were mainly women (16). In a previous study, excitatory neurons of the somatosensory cortex were hyperexcitable in interictal women with frequent migraine attacks, in this context, stronger PAG-FC with the somatosensory cortex might make the female brain more susceptible for migraine attacks (34). Moreover, female migraineurs have thickened somatosensory cortex compared to healthy controls, but again, male results are missing (35).

Previously, in healthy females, PAG showed increased pain-induced connectivity with SMA compared to men (13). SMA also has a higher likelihood of neuromagnetic activation in ictal female migraineurs during finger moving task that produces cortical hyperexcitability (36). Our results indicate stronger resting-state connectivity between PAG and SMA in pain-free female migraineurs compared to males. The stronger PAG-FC with SMA may strengthen the notion of higher cortical hyperexcitability of the female brain between attacks.

In a previous study, healthy women compared to men also have stronger resting-state PAG-FC with MCC (37), involved in cognitive, affective, and attentional aspects of pain (38). Another resting-state connectivity analysis of subgenual anterior cingulate cortex (sgACC) revealed stronger connectivity between sgACC and PAG and MCC in healthy women compared to men indicating that women have a greater descending antinociceptive modulation contributing to more effective pain habituation compared to men (39). In migraine, a pain-induced increased activity of MCC was observed (40), however, sex differences were not assessed. Our results indicate a stronger resting-state PAG-FC with MCC in pain-free female migraineurs compared to males that might contribute to the sex phenotype of migraine, but based on previous findings in healthy samples it might represent a sex-specific brain pattern rather than a disease-specific one (10).

We also found a stronger PAG-FC with visual cortex including cuneus, calcarine and superior occipital gyrus. In a previous study, these regions had a stronger pain-induced PAG-FC in migraineurs compared to controls (41), and our results on sex differences might suggest that in migraine this increased connectivity might contribute to the increased interictal sensory sensitivity for females (42) that could lead to an increased vulnerability for migraine attacks (43). Impaired attentive processing of visual stimuli was also observed in female migraineurs compared to males suggesting that neurocognitive processing is affected by migraine in females (44).

Furthermore, we have to note that PAG also plays a critical role in autonomic processes, behavioural, cardiovascular, and respiratory responses to stressors and the different connectivity of PAG subregions may regulate different physiological functions (45, 46). The dysfunction of PAG is also implicated in anxiety (47) and depression (48), which disorders are known to be sex-differentiated and also comorbid with migraine (49).

### Average Pain Intensity Shows Interaction With Sex on PAG-FC

In accordance with the general notion that migraine intensity is similar in males and females (50), our participants did not differ in average pain intensity. However, the average pain intensity of migraine attacks interacted with sex on PAG-FC in our analysis. Interestingly, increased connectivity between PAG and the angular, superior parietal, middle temporal, and superior occipital gyri correlated with higher average pain intensity only in male migraineurs.

In patients with fibromyalgia, increased activity of the angular gyrus and its stronger connectivity with pain- and analgesia-related brain areas correlated with pain intensity analgesia after listening to music (51). In addition, the angular gyrus may exert an analgesic effect by top-down pain control during distraction or placebo analgesia (51). Indeed it has been suggested that problem-focused avoidance (cognitive and behavioural distraction) is the most frequently reported pain coping strategy for men (52).

The superior parietal, middle temporal, and occipital gyri are also involved in pain processing, their activity correlated with subjective pain in a study using a thermal heat task (53). Interestingly, these regions positively mediated the relationship between pain intensity and reported pain, however other areas such as the primary motor and the activity of the sensory cortex were uniquely related to temperature but not to pain report (53). In the study of Atlas et al., sex differences in the activity of pain mediator regions were not assessed, however, the majority of participants were male. Those previous studies in parallel with our result suggest an increased sensory sensitivity in female migraineurs (indicated by stronger PAG-FC with somatosensory cortex), meanwhile intensified subjective pain experience in male migraineurs (denoted by the correlation of average pain intensity and PAG-FC).

The PAG-FC with the above-mentioned regions may be also implicated in pain-related unpleasantness (3). Men generally show greater brain activation in pain-activated regions for both low and high pain stimuli compared to women (54). Another study demonstrated that pain intensity augments the sex differences in PAG-FC: increasing pain intensity is associated with enhanced PAG connectivity with emotion-processing areas (amygdala, caudate, and putamen) in males (13). In addition, only men showed a placebo response to pain unpleasantness, and a larger stress reduction was observed in the absence of pain among men compared to women (55). The connexion of PAG with areas of the subjective experience of pain associated with more intense migraine attacks in men in our study may suggest that they use distraction as a possible pain coping mechanism (52). This finding raises the attention to male-specific neuropathology of migraine that is less known. Migraine is underdiagnosed among males possibly because they are less willing to report pain and usually avoid going to the doctor to pretend the image of “brave man” over against “emotional woman” (56) and they are willing to use cognitive and behavioural distraction more and have higher perceived self-efficacy compared to women (57). Nonetheless, our result suggests that interindividual differences in general perceived subjective pain intensity in males might be associated with connectivity of PAG with angular, superior parietal, middle temporal, and occipital gyri.

### Migraine-Related Disability Correlated With PAG-FC but Did Not Interact With Sex

In general, women are more likely to report severe headache-related disability compared to men (58). In our study, migraine-related disability did not differ between sexes. It was related to the FC of PAG with superior medial part of frontal gyrus (more precisely dorsomedial prefrontal cortex: dmPFC) connectivity. Specifically, higher migraine-related disability associated with decreased PAG-dmPFC connectivity indicates a weaker top-down pain control (32) without sex differences. The PFC-PAG circuitry is involved in the emotional modulation of pain (59), thus the lower PAG-FC with dmPFC in our study may suggest a weaker pain inhibition in parallel with higher migraine-related disability. A recent animal study demonstrated that the dmPFC and PAG pathway is involved in pain threshold maintenance and antianxiety behaviour, and the deactivation of dmPFC induces negative emotions and may lead to pain chronification (60). Our results are in concordance with this observation; however, it is not clear whether the decreased PAG-dmPFC connectivity is the cause or the consequence of increased migraine-related disability. There is a general notion about higher migraine-caused disability in women than men, but according to our questionnaire and fMRI results, the neuronal background of migraine disability did not show sex differences.

### Migraine Frequency Did Not Correlate With PAG-FC

In general, female migraineurs have more frequent migraines than males (61). In our study, there was no difference in migraine frequency between female and male migraine groups. Migraine frequency did not correlate with PAG-FC and we found no sex differences. In previous studies, migraine frequency showed association with PAG-FC for example with SMA, prefrontal, and somatosensory cortex (16, 41) that we did not detect. The direct comparison with these studies is difficult because they investigated mixed migraine patients with and without aura, or patients on daily medication with high migraine frequency. However, we have examined a homogenous episodic migraine population without aura which is also a strength of the study.

## Limitations

There are some limitations of our study that we should mention in this study. First, we have a small sample size in each studied group. Second, we did not have healthy control groups. Instead, we used literature results to compare our results to the sex differences in non-migraine subjects. Third, the fMRI data acquisition was assessed in two different types of MR scanners. However, we did not detect any scanner-related effects. The resolution of MR scanners could be also a potential limitation because scanning brainstem structures such as PAG is highly affected by physiological noise and the anatomical complexity of small this small region (62). In a complex preprocessing pipeline we tried to address these challenges (refer to Supplementary Figure 1). In our study, only the two sides of the PAG were used as seed regions, however, PAG connectivity may differ between subregions as well (45, 63). Sex differences between subregional connectivity would be an interesting direction for future studies.

Finally, female hormones might affect pain processing and PAG function (3, 17) thus we investigated the effect of oral contraceptives and menstrual cycle on PAG-FC. Since no differences were found, we analysed female participants together. However, in the future, it would be interesting to know whether sex hormones affect differently the functional connectivity of PAG in a larger number of migraineurs.

## Conclusion

In this study, a stronger PAG functional connectivity with regions of ascending pain pathways in female migraineurs indicates increased brain excitability that might be a risk factor for migraine. Therefore, the PAG network may contribute to greater sensitivity and migraine vulnerability distinctively in women. However, the average pain intensity of migraine attacks exhibits stronger PAG connectivity with regions involved in the subjective experience of pain in males which may represent a consequence of increased subjective pain experience. Based on this observation, male-specific mechanisms of migraine should gain more attention. The association between other migraine characteristics and PAG connectivity seems to represent general pain-related features of migraine without sex differences. PAG connectivity shows alterations in association with migraine-related disability suggesting a decreased descending pain control. In conclusion, we were able to identify general and sex-specific differences in PAG functional connectivity among migraineurs, and thereby, show with novel results how the “migraine brain” may be differently affected in females and males even in the interictal state.

## Author's Note

Preliminary data from this study were presented at the following event: 34th ECNP Congress 2–5 October 2021, Lisbon, Portugal poster presentation.

## Data Availability Statement

The datasets presented in this article are not readily available because they contain information that could compromise the privacy of research participants. Requests to access the datasets should be directed to juhasz.gabriella@pharma.semmelweis-univ.hu.

## Ethics Statement

The studies involving human participants were reviewed and approved by Scientific and Research Ethics Committee of the Medical Research Council (Hungary). The patients/participants provided their written informed consent to participate in this study.

## Author Contributions

The study was designed and conceived by GJ and GK. ES, NK, and AG were responsible for subject recruitment and data collection. Data analysis was performed by KG with assistance from GJ, GK, CSA, and ME. GB, DD, DB, and GJ contributed to the interpretation to the data. KG, DB, and GJ wrote the first draught of the manuscript. All authors contributed to the article and approved the submitted version.

## Funding

This study was supported by the Hungarian Academy of Sciences (MTA-SE Neuropsychopharmacology and Neurochemistry Research Group); the Hungarian Brain Research Program (Grants: 2017-1.2.1-NKP-2017-00002; KTIA_13_NAPA-II/14); the National Development Agency (Grant: KTIA_NAP_13-1-2013- 0001); by the Hungarian Academy of Sciences, Hungarian National Development Agency, Semmelweis University and the Hungarian Brain Research Program (Grant: KTIA_NAP_13-2- 2015-0001) (MTA-SE-NAP B Genetic Brain Imaging Migraine Research Group); by the Thematic Excellence Programme (Tématerületi Kiválósági Program, 2020-4.1.1.-TKP2020) of the Ministry for Innovation and Technology in Hungary, within the framework of the Neurology and Translational Biotechnology thematic programmes of the Semmelweis University; by the National Research, Development and Innovation Office, Hungary (2019-2.1.7-ERA-NET-2020-00005), under the frame of ERA PerMed (ERAPERMED2019-108); and by the ÚNKP-20-3-II-SE-51 New National Excellence Program of the Ministry for Innovation and Technology from the source of the National Research, Development and Innovation Fund. The sponsors had no role in the design of the study, in the collection, analysis, interpretation of data, and in the writing of the manuscript.

## Conflict of Interest

The authors declare that the research was conducted in the absence of any commercial or financial relationships that could be construed as a potential conflict of interest.

## Publisher's Note

All claims expressed in this article are solely those of the authors and do not necessarily represent those of their affiliated organizations, or those of the publisher, the editors and the reviewers. Any product that may be evaluated in this article, or claim that may be made by its manufacturer, is not guaranteed or endorsed by the publisher.
