# Supplementary material for: Sex Differences of Periaqueductal Grey Matter Functional Connectivity in Migraine
Source: Front Pain Res (Lausanne). 2021 Nov 30;2:767162. doi: 10.3389/fpain.2021.767162 (PMC8915645; doi:10.3389/fpain.2021.767162)
Supplement: Supplementary file 1 [file Data_Sheet_1.docx]

Supplementary material

**Supplementary table 1. Participants’ characteristics according to scanner site**

|  | Philips Achieva | Siemens MAGNETOM | Test statistics (U) | *p* |
| --- | --- | --- | --- | --- |
| Number of participants | 16 | 16 |  |  |
| Age (years) | 27.69±5.42 | 31.42±6.26 | 86.0 | 0.119 |
| Migraine frequency /month | 2.89±2.25 | 3.25±3.17 | 123.5 | 0.867 |
| MIDAS total score | 11.79±12.60 | 8.0±7.41 | 102.0 | 0.697 |
| Average pain intensity (MIDAS-B) | 5.75±1.67 | 5.19±1.76 | 87.0 | 0.313 |
| Oral contraceptives (OC) | 3 females | 3 females |  |  |
| Menstrual cycle  (those who were not on OC) | 4 females in luteal;  1 female in follicular | 2 females in luteal;  3 females in follicular |  |  |

*Note* Data are expressed as mean value ± SD. The p values are based on Mann-Whitney U tests. MIDAS: Migraine Disability Assessment questionnaire, MIDAS total score was calculated as a measure of migraine related disability, MIDAS-B refers to the average pain intensity of migraine attacks.

**Supplementary table 2. Brain regions showing a significantly different PAG–FC between female and male migraineurs**

| Cluster Size  (voxel) | Region | Peak coordinates | | | Peak *F*-value |
| --- | --- | --- | --- | --- | --- |
|  |  | x | y | z |  |
| *Left PAG* | | | | | |
| 217 | R Postcentral gyrus | 60 | -14 | 42 | 32.65 |
|  | R Precentral gyrus | 62 | 2 | 38 | 21.36 |
| 124 | L Inferior parietal gyrus | -56 | -20 | 50 | 30.18 |
|  | L Postcentral gyrus | -54 | -28 | 54 | 21.93 |
| 125 | R Precentral gyrus | 40 | -10 | 62 | 29.64 |
| *Right PAG* | | | | | |
| 579 | R Postcentral gyrus | 60 | -14 | 42 | 39.52 |
|  | R Precentral gyrus | 40 | -12 | 64 | 35.65 |
| 117 | L Calcarine | -20 | -70 | 14 | 35.26 |
|  | L Cuneus | -14 | -76 | 24 | 32.96 |
|  | L Superior occipital gyrus | -14 | -86 | 40 | 22.04 |
| 150 | L Postcentral gyrus | -54 | -20 | 52 | 27.87 |
| 90 | L Supplementary motor area | -8 | -12 | 48 | 22.81 |
|  | L Midcingulate cortex | -4 | -6 | 38 | 20.24 |

*Note* Reported results are significant at cluster-level pFWE<0.05. Coordinates are in Montreal Neurological Institute (MNI) space.

R: right hemisphere, L: left hemisphere

**Supplementary figure 1. Imaging preprocessing steps**
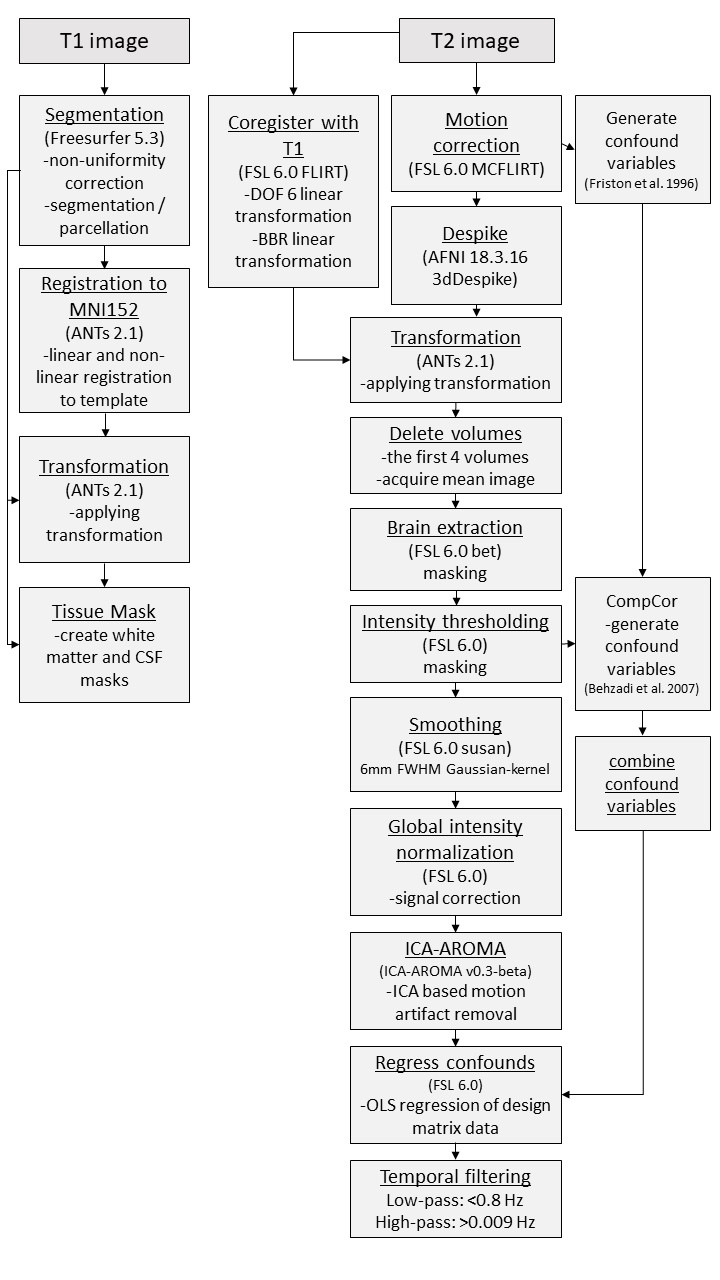


**Supplementary figure 2. Seed regions**

Seed regions were defined after Mainero et al. left PAG (red) = -2; -28; -6; right PAG (green) = 4; -28; -6; coordinates in MNI space with a 3mm radius.

**
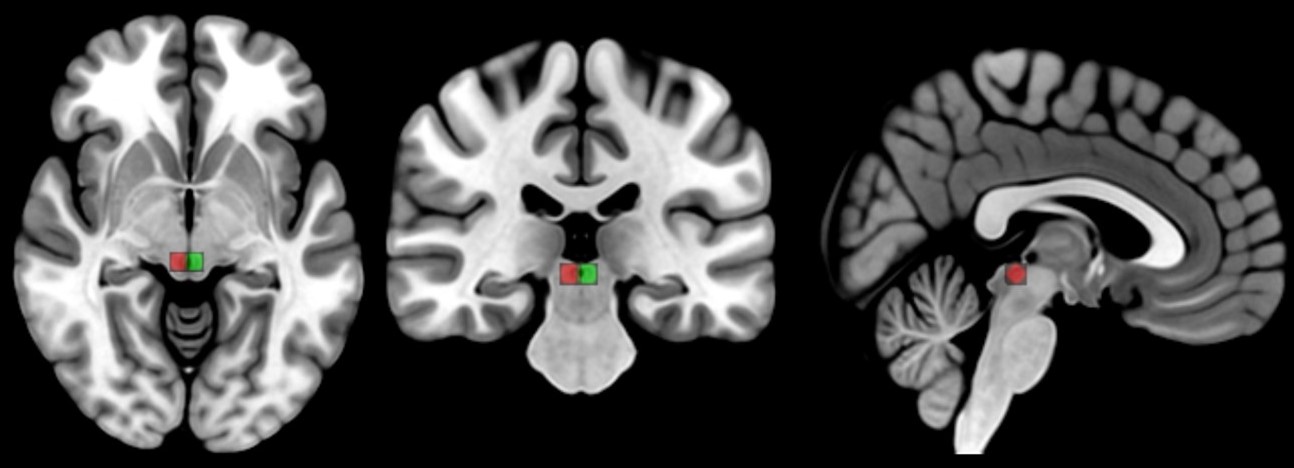
**

*Reference*: Mainero C, Boshyan J, Hadjikhani N. Altered functional magnetic resonance imaging resting-state connectivity in periaqueductal gray networks in migraine. Ann Neurol (2011) 70(5):838-45. Epub 2011/12/14. doi: 10.1002/ana.22537.
